# A Fibroblastic Reticular Cell Tumour Arising in the Oral Cavity: A Case Report and Review of the Literature

**DOI:** 10.1007/s12105-022-01496-9

**Published:** 2022-11-07

**Authors:** Hannah Walsh, Daniel Brierley, Alan Patterson, Malee Fernando

**Affiliations:** 1 School of Clinical Dentistry, University of Sheffield, Sheffield S10 2TA, Sheffield, UK; 2 Department of Oral and Maxillofacial Surgery, Rotherham General Hospital, Moorgate Road, Rotherham S60 2UD, Rotherham, UK; 3 Haemato-oncology Diagnostic Services (HODS), Department of Histopathology, Sheffield Teaching Hospitals, Royal Hallamshire Hospital, Glossop Road, Sheffield S10 2JF, Sheffield, UK

**Keywords:** Oral pathology, Tongue, Neoplasms, Dendritic cells, Immunohistochemistry, Diagnosis(oral)

## Abstract

**Background:**

Tumours of dendritic or histiocytic lineage are amongst the rarest tumours and probably account for < 1% of tumours affecting the lymph nodes or soft tissue. Because several of these entities were poorly recognised until recently, the true incidence is not determined.

**Methods:**

We present what we believe is the first reported case report of a fibroblastic reticular cell tumour arising in the oral cavity as well as reviewing the current literature regarding this rare subset of tumours.

**Results:**

We discuss the clinical and histopathological findings of our reported case and examine the literature regarding this entity. We discuss the key differential diagnoses to consider when making this diagnosis.

**Conclusion:**

Histiocytic and dendritic cell derived tumours are exceptionally rare within the head and neck region although a number of these tumours have been reported within the oral cavity. We present what we believe is the first reported case of a fibroblastic reticular cell tumour arising within the oral cavity.

## Introduction

Tumours of dendritic or histiocytic origin are exceedingly rare, accounting for < 1% of tumours affecting lymph nodes or soft tissues. Within this case report we discuss what we believe to be the first fibroblastic reticular cell tumour arising in the oral cavity to be documented within the literature as well as discussing the current literature and the differential diagnoses.

## Case Report

A 59-year-old male was referred to his local Oral and Maxillofacial department by his general dental practitioner with a three-week history of painful bilateral tongue lesions with ulceration. The patient complained of pain radiating to the maxilla and mandible which was present when swallowing and eating and he reported weight loss of 10 kg over the previous 6 months. The patient’s medical history included; type II diabetes, diabetic nephropathy, chronic obstructive pulmonary disease, hypercholesterolaemia, osteoarthritis and vitamin D deficiency which were managed by multiple medications. He was an ex-smoker, previously smoking 20 cigarettes a day, and consumed 10 units of alcohol per week.

**Fig. 1:**
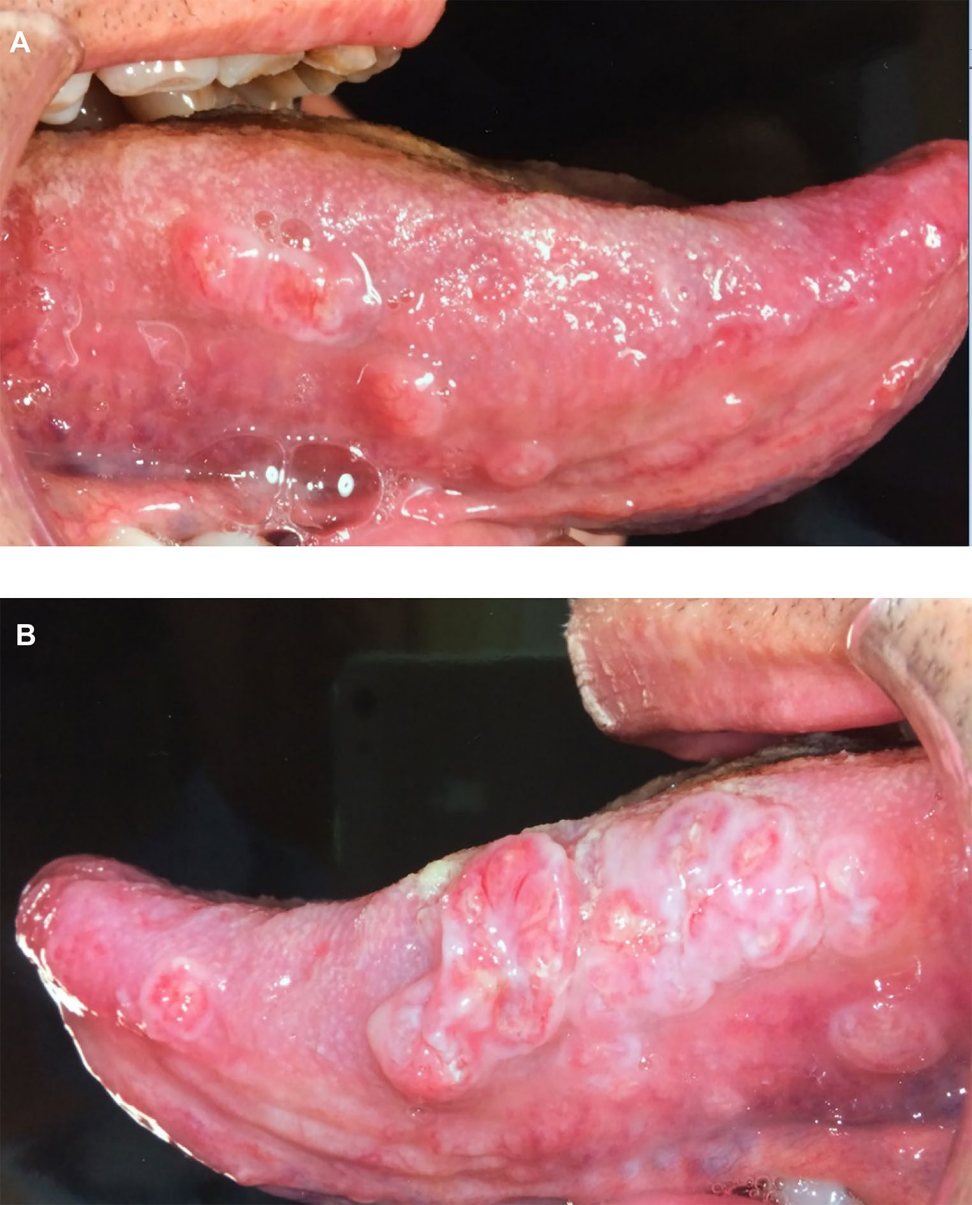
Clinical presentation of tongue lesions **A**: Right lateral border of tongue **B**: Left lateral border of tongue

Clinical examination demonstrated multiple small, indurated, tender nodular lesions on the lateral borders of the tongue (Fig. 1). An incisional biopsy was sent for histological examination.

**Fig. 2:**
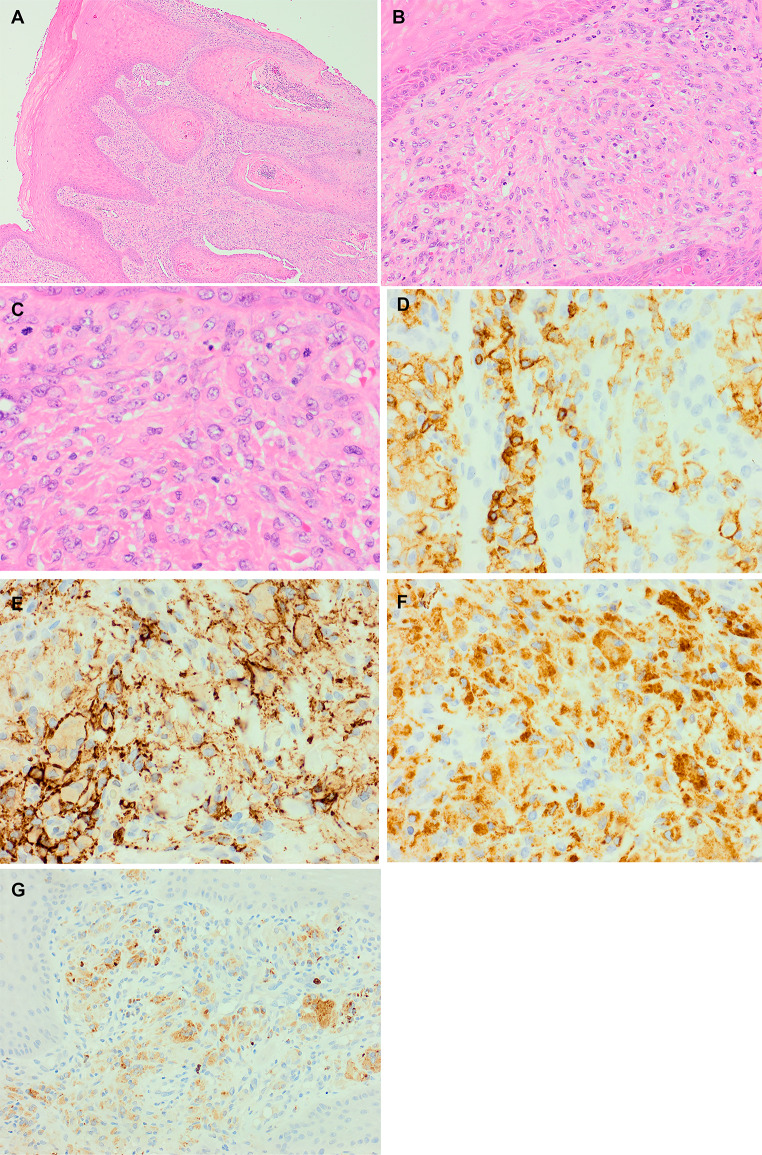
Histological findings on H&E showing; **A**: overlying pseudoepitheliomatous hyperplasia without dysplasia, **B**: the tumour abutting but not invading the epithelium, **C**: high power view of the tumour cells displaying the irregular nuclear margins without nucleoli or intranuclear grooves and scattered mitoses; positive staining with IHC; **D**: CD45, **E**: CD14, **F**: CD68 and **G**: Lysozyme.

Histological examination showed pseudoepitheliomatous hyperplasia without dysplasia. There was a histiocytic infiltrate within the sub-epithelial tissue seen to abut but not invade the epithelium (Fig. 2). The infiltrate was composed mainly of ovoid mononuclear cells with abundant eosinophilic cytoplasm and a few admixed multinucleated forms. There were occasional neoplastic spindled forms in continuity with the ovoid cells. The nuclei were open with irregular nuclear margins but without nucleoli or intranuclear grooves. Scattered mitoses were identified although no signs of apoptosis or necrosis were present. The neoplastic cells were admixed with eosinophils and few lymphocytes. No evidence of lymphoma or amyloid deposition was evident in the background. The immunohistochemical profile is summarised in the subsequent table.

The IHC findings confirmed the histiocytic nature of the neoplasms and the lesion was subtyped as a Fibroblastic reticular cell tumour (also known as cytokeratin-positive interstitial reticulum cell tumour).

## Discussion

Malignancies of dendritic or histiocytic origin are exceedingly rare, accounting for less than 1% of all neoplasms arising in soft tissue or lymph nodes [1]. Tumours are divided into two groups based upon their derivation from either bone marrow precursors or either mesenchymal or stromal derived dendritic cells. Interdigitating dendritic cell sarcoma (IDCS), Langerhans cell histiocytosis (LCH) and histocytic sarcoma (HS) are of bone marrow linage whereas intermediate dendritic cell sarcoma (INDCS), follicular dendritic cell sarcoma (FDCS), disseminated juvenile xanthogranuloma (DJX) and fibroblastic reticular cell tumours (FRCTs) are thought to be related to either stromal-derived dendritic cell or cells of mesenchymal origin [2, 3].

FRCTs are thought to be derived from mesenchymal stem cells and express smooth muscle and histiocytic markers. They primarily occur in lymph nodes but lesions have also been identified in the liver, spleen, lung and soft tissue [1], although extra-nodal primary presentation is rare [4]. The most commonly involved nodes are the cervical and mediastinal nodes [1]. To the best of our knowledge this is the 24th documented FRCT in the literature with none of the other documented cases involving the oral cavity [4–8]. Other dendritic cell sarcomas reported in the oral cavity include 9 FDCSs [8] and 1 IDCS [9].

The expression of cytokeratins in an undifferentiated tumour can cause diagnostic problems as it may lead to a diagnosis of metastatic carcinoma. Whilst the lack of true epithelial morphology, reticular pattern of infiltration and hybrid immunophenotype with co-expression of cytokeratins and mesencyhmal markers help distinguish these neoplasms from metastatic carcinoma [10], the key to the diagnosis is the knowledge of this entity.

The histological morphology and IHC findings were key to achieving the final diagnosis for this case and as always the immunophenotype needs to be interpreted in relation to the morphology of the cells. The tumour cells had a predominantly epithelioid morphology and spindling was only focal. FDCS are mainly spindled and express FDC markers – which were conspicuously absent in this tumour. FDCS are typically positive for CD21, CD23, CD35, fascin and are variably positive with CD68, with the first two being the more specific markers [1]. This tumour did not express CD4, CD21 or fascin and had only variable expression with CD68. The expression of broad spectrum cytokeratin is usually not seen in the non-FRCT tumours within the histiocytic spectrum of tumours. SMA, desmin and CD123 are not lineage specific and the morphology and phenotype did not support a tumour with smooth muscle differentiation or a BPDCT. The patient did not have CMML either, which is important when cells with CD123 are identified.

Other differential diagnoses of a Langerhan cell sarcoma (LHS), IDCS or INDCS were considered and excluded based upon tumour morphology and IHC. LHS express S100, langerin and CD1a [11], with our case being negative for both langerin and S100. IDCS are positive with CD4, CD68, fascin, S100 and show variable positivity with CD45, with our case being positive for CD45 and CD68 but negative for the other IHC markers mentioned [1]. INDCS are positive for CD4, fascin, S100 and show variable expression with CD68, whereas our case was negative for CD4, fascin and S100 [1]. Although some of the IHC findings raised the possibility of a HS, as the tumour was also positive for CD68 and lysozyme, the morphology was not typical as the cells found in HS are large to round with focal spindling [1]. Similarly, the morphological appearance did not support the possibility of a plasmacytoid dendritic cell infiltrate.

**Table 1:** Summary of IHC findings

Antibody	Reactivity
CD14	+
CD45	+
CD68 (PG-M1)	+
CD123	+
Lysozyme	+
CD163	+ (patchy)
AE1/AE3	+/-(in a dendritic pattern)
CD13	+/-
CD35	+/-
S100	-
CAM 5.2	-
SMA	+/-
Desmin	-
CD21	-
MNF116	-
MPO	-
Langerin	-
ALK1	-
Fascin	-
CD4	-
CD56	-
CD138	-

Key: + (positive), - (negative), +/- (variable expression)

**Table 2:** Summary of the clinical, histopathological and immunophenotypes of the differential diagnoses.

Tumour	FRCT	IDCS	INDCS	FDCS	LHS
Clinical Presentation	Can occur in lymph nodes, spleen or soft tissue.	Most commonly found within a solitary lymph node, but reports of extranodal presentation.	Papular skin lesions.	Mainly nodal involvement although can be extra-nodal in GI tract & skin.	Mainly extra nodal including bone and skin. Often multifocal.
Histological Presentation	Similar to FDCS or interdigitating dendritic cell sarcoma but lack their IHC pattern.	Paracortical lesional distribution within nodes. Neoplastic cells have ovoid nuclei and the cells are arranged in fasicles. Low mitotic count.	Similar appearance to Langerhans cells - nuclear grooves and clefts.	Tumour has a whorled pattern with the neoplastic cells having eosinophilic cytoplasm and ovoid nuclei.	Tumour cells show prominent nuclear pleomorphism, with clumped chromatin and inconspicuous nucleoli. High mitotic count.
IHC Findings (positive stains)	Vimentin, SMA	CD4, CD68, Fascin, S100	CD4, Fascin, S100	CD4, CD21,CD23, CD35, Fascin	S100, Langerin, CD1a
IHC Findings (variable stains)﻿	SMA, Desmin, cytokeratin (dendritic pattern), CD68	CD45, Lysozyme	CD68	CD68, S100, EMA	
IHC Findings (negative stains)	CD1a, S100, CD21, CD35	CD1a, Langerin, CD21, CD23, CD35, Myeloperoxidase, CD34, CD30, EMA, cytokeratins	CD1a	CD1a, lysozyme, CD34, CD3, CD30, Myeloperoxidase	CD21, CD35, CD23

The lesion was diagnosed as a FRCT and further clinico-pathological correlation was recommended to exclude a marrow infiltrate of neoplastic monocytic lineage. A bone marrow trephine biopsy confirmed an incidental finding of a concomitant Plasma cell myeloma (~ 75% plasma cells, consistent with a Plasma cell myeloma, no histiocytic infiltrate). Imaging investigations of a CT scan of the chest, abdomen and pelvis did not identify any lytic bone lesions or intra-abdominal lymph nodes, and an MRI scan of the head demonstrated subtle asymmetrical thickening of left tongue base (piriform fossa) which was deemed to be non-specific. No lytic bone lesions or focal mass lesions were identified.

With the limited number of cases reported in the literature, the most appropriate treatment is not yet certain. From the literature patients with localised disease are best treated primarily with surgery [1]. There is limited evidence to support the use of adjuvant radiotherapy alongside surgery in patients with localised disease [1, 4] and some authors suggest that chemotherapy has no role in local disease [1]. One study has found that the two-year survival in patients with local disease is 85.7% [8], whereas all patients with distant disease died within two years [8].

In the case we discuss, following the patient’s diagnoses of myeloma and FRTC of the tongue, this patient was treated by the haematology department with VTD chemotherapy – a combination of Velcade, Thalidomide and Dexamethasone. The patient reported some improvement to the tongue lesions whilst receiving VTD. The patient had 4 cycles of chemotherapy over 4 months and then had an autograft stem cell transplant. One year post-transplant the patient was reviewed by the haematology department who reported that his myeloma was undetectable and the patient was managing to eat and drink without problems. Unfortunately the patient died shortly after this review, 18 months following his diagnoses. He was admitted to hospital experiencing shortness of breath and was found to have pneumonia. He passed away shortly after admission following a myocardial infarction.

In summary, we present what we believe to be the first reported case in the literature of a FRCT arising in the oral cavity. The rarity of these lesions, their diagnostic challenges and the limited data regarding treatment have been highlighted. The details we have provided regarding the case presented will hopefully help to increase awareness and improve knowledge amongst the pathology community regarding this rare group of tumours and this extremely rare subtype.

## Data Availability

**(data transparency)**: This case has been presented as a poster presentation at the BSOMP Annual Scientific Meeting 2018. This publication is not for consideration and has not been submitted elsewhere.

## Code Availability

**(software application or custom code) Data availability statements** : Not applicable for this publication.

## Declarations

### Additional Declarations for Articles in life Science Journals that Report the Results of Studies Involving Humans and/or Animals:

Not applicable for this publication.
